# The Evolution of Sex Ratio Distorter Suppression Affects a 25 cM Genomic Region in the Butterfly *Hypolimnas bolina*

**DOI:** 10.1371/journal.pgen.1004822

**Published:** 2014-12-04

**Authors:** Emily A. Hornett, Bruce Moran, Louise A. Reynolds, Sylvain Charlat, Samuel Tazzyman, Nina Wedell, Chris D. Jiggins, Greg D. D. Hurst

**Affiliations:** 1 Institute of Integrative Biology, University of Liverpool, Liverpool, United Kingdom; 2 Department of Zoology, University of Cambridge, Cambridge, United Kingdom; 3 Laboratory of Biometry and Evolutionary Biology, CNRS - University Lyon, Villeurbanne, France; 4 Faculty of Life Sciences, University College London, London, United Kingdom; 5 Theoretical Biology, ETH Zürich, Zürich, Switzerland; 6 Centre for Ecology and Conservation, University of Exeter, Penryn, United Kingdom; Fred Hutchinson Cancer Research Center, United States of America

## Abstract

Symbionts that distort their host's sex ratio by favouring the production and survival of females are common in arthropods. Their presence produces intense Fisherian selection to return the sex ratio to parity, typified by the rapid spread of host ‘suppressor’ loci that restore male survival/development. In this study, we investigated the genomic impact of a selective event of this kind in the butterfly *Hypolimnas bolina*. Through linkage mapping, we first identified a genomic region that was necessary for males to survive *Wolbachia*-induced male-killing. We then investigated the genomic impact of the rapid spread of suppression, which converted the Samoan population of this butterfly from a 100∶1 female-biased sex ratio in 2001 to a 1∶1 sex ratio by 2006. Models of this process revealed the potential for a chromosome-wide effect. To measure the impact of this episode of selection directly, the pattern of genetic variation before and after the spread of suppression was compared. Changes in allele frequencies were observed over a 25 cM region surrounding the suppressor locus, with a reduction in overall diversity observed at loci that co-segregate with the suppressor. These changes exceeded those expected from drift and occurred alongside the generation of linkage disequilibrium. The presence of novel allelic variants in 2006 suggests that the suppressor was likely to have been introduced via immigration rather than through *de novo* mutation. In addition, further sampling in 2010 indicated that many of the introduced variants were lost or had declined in frequency since 2006. We hypothesize that this loss may have resulted from a period of purifying selection, removing deleterious material that introgressed during the initial sweep. Our observations of the impact of suppression of sex ratio distorting activity reveal a very wide genomic imprint, reflecting its status as one of the strongest selective forces in nature.

## Author Summary

The sex ratio of the offspring produced by an individual can be an evolutionary battleground. In many arthropod species, maternally inherited microbes selectively kill male hosts, and the host may in turn evolve strategies to restore the production or survival of males. When males are rare, the intensity of selection on the host may be extreme. We recently observed one such episode, in which the population sex ratio of the butterfly *Hypolimnas bolina* shifted from 100 females per male to near parity, through the evolution of a suppressor gene. In our current study, we investigate the hypothesis that the strength of selection in this case was so strong that the genomic impact would go well beyond the suppressor gene itself. After mapping the location of the suppressor within the genome of *H. bolina*, we examined changes in genetic variation at sites on the same chromosome as the suppressor. We show that a broad region of the genome was affected by the spread of the suppressor. Our data also suggest that the selection may have been sufficiently strong to introduce deleterious material into the population, which was later purged by selection.

## Introduction

In 1930, Fisher noted that the strength of selection on the sex ratio was frequency dependent, echoing earlier findings of Düsing [1], [2]. As a well-mixed outbreeding population progressively deviates from a 1∶1 sex ratio, selection on individuals to restore the sex ratio to parity becomes stronger. In natural animal populations, a common cause of population sex ratio skew is the presence of sex ratio distorting elements, in the form of either sex chromosome meiotic drive [3], or cytoplasmic symbionts [4]. In some cases, these elements can reach very high prevalence, distorting population sex ratios to as much as 100 females per male [5], and producing intense selection for restoration of the individual sex ratio to 1 female per male. The most common consequence of this selection pressure is the evolution of systems of suppression – host genetic variants that prevent the sex ratio distorting activity from occurring. Suppressor factors are known for a wide range of cytoplasmic symbionts and meiotic drive elements [3], [6], [7].

The evolution of suppression of *Wolbachia* induced male-killing activity in the butterfly *Hypolimnas bolina* represents a compelling observation of intense natural selection in the wild. Female *H. bolina* can carry a maternally inherited *Wolbachia* symbiont, *w*Bol1, which kills male hosts as embryos [8]. The species also carries an uncharacterised dominant, zygotically acting suppression system that allows males to survive infection [6]. Written records and analysis of museum specimens indicate this symbiont was historically present, and active as a male-killer, across much of the species range, from Hong Kong and Borneo through to Fiji, Samoa and parts of French Polynesia [9]. Evidence from museum specimens also indicates that host suppression of male-killing had a very restricted incidence in the late 19^th^ century, with infected male hosts (the hallmark of suppression) being found in the Philippines but not in other localities tested. By the late 20^th^ century, suppression of male-killing was found throughout SE Asia, but not in Polynesian populations where the male-killing phenotype remained active [10]. The most extreme population was that of Samoa, where 99% of female *H. bolina* were infected with male-killing *Wolbachia*, resulting in a sex ratio of around 100 females per male within the population [5]. However, following over 100 years of stasis on Samoa, the rapid spread of suppression of male-killing activity of the bacterium was finally observed between 2001 and 2006, restoring both individual and population sex ratio to parity [11].

When strong selection occurs at a locus, it is expected to leave a genomic imprint beyond the target of selection, as a result of genetic hitch-hiking. A neutral (or even deleterious) variant that is initially present in the haplotype in which the favoured allele arose (i.e. is linked to the site of selection), will also increase in frequency [12]. When selection is very strong, the frequency of linked variants may increase across a broad genomic region [13]. Importantly, the extent of the chromosome over which this effect will occur depends on the selection pressure in the first few generations; before recombination has broken down associations between the target of selection and linked variants. Where sex ratio distorters are common, the selection pressure in these first generations may be very strong indeed (before the sex ratio becomes less biased through spread of the suppressor). It is thus likely that selection on the sex ratio will influence linked material over a broader genomic region compared to many other selective regimes. That is, the episode of selection is likely to have a very wide genomic impact.

In this paper, we first mapped a genomic region in SE Asian butterflies that was required for male survival in the presence of *Wolbachia*. We then investigated the impact of the recent spread of the suppressor in Samoa on the pattern of variation around this region. To this end, we initially developed theory to predict the impact of suppressor spread on linked genetic variation. We then directly observed changes in the frequency of genetic variants surrounding the suppressor locus by comparing the pattern of genetic variation in *H. bolina* specimens collected in Samoa before (2001) and after the selective sweep (2006 and 2010). By examining post-sweep samples at two time points we were additionally able to track allele frequency changes following the initial sweep. The data revealed changes in the pattern of genetic variation over a 25 cM region surrounding the suppressor locus. We further suggest that the suppressor was probably derived by immigration, and that the sweep may have introduced deleterious material that was subsequently subject to purifying selection.

## Results

### Location of a region required for male survival in the *H. bolina* genome

*Hypolimnas bolina* has 31 chromosomes and a total genome size of 435 MB [14], [15]. Previous work established that the rescue of male zygotes from *Wolbachia* induced killing was dominant, and potentially a single locus trait [6]. Genetic markers spanning the genome were developed using a targeted gene approach informed by conservation of synteny in Lepidoptera, with the sequence of *H. bolina* orthologs obtained through Roche 454 transcriptome sequencing (see Methods and Materials, NCBI SRA accession: SRP045735). These markers were then tested for co-segregation with suppression in order to identify the linkage groups associated with male host survival. Female butterflies from South East (SE) Asia that carried both *Wolbachia* and the suppressor allele, were crossed with males from the French Polynesian island Moorea (where suppression is absent). The resulting F1 daughters (who inherited *Wolbachia* from their SE Asian mother) were then backcrossed to Moorea males to create a female-informative family for identification of loci linked to the suppressor. The absence of recombination in female Lepidoptera means that a SE Asia allele on any chromosome that is necessary for male survival will be present in all of the surviving sons of this female (as if they lack it, they die), but this allele will show normal 1∶1 segregation in her daughters (S1 Figure). Initially 10 loci from across the genome were screened. Of these, one locus orthologous to sequence on chromosome 25 in the moth *Bombyx mori* showed this unusual pattern of inheritance. For this locus, all 16 sons carried the same maternal allele of SE Asia origin while 8 daughters showed Mendelian segregation (probability of observing this pattern of segregation in sons on the null hypothesis of no association = (1/2)^16^: p<0.0001).

We then obtained an additional 11 markers in this linkage group. Candidates were identified initially via synteny to *B. mori*, and then confirmed as showing co-segregation with the original marker and as being associated with male survival, in the female-informative family. In this way, a suite of 12 suppressor-linked markers (A-L) were developed, all of which followed the presumed pattern of inheritance of the suppressor - that of presence in all 16 sons and half of the daughters. The remaining 9 non-suppressor-linked markers (M-U), representing 8 separate linkage groups, were developed to investigate potential genome-wide effects. Marker information and accession numbers are given in S1 Table and S2 Table.

**Figure 1:**
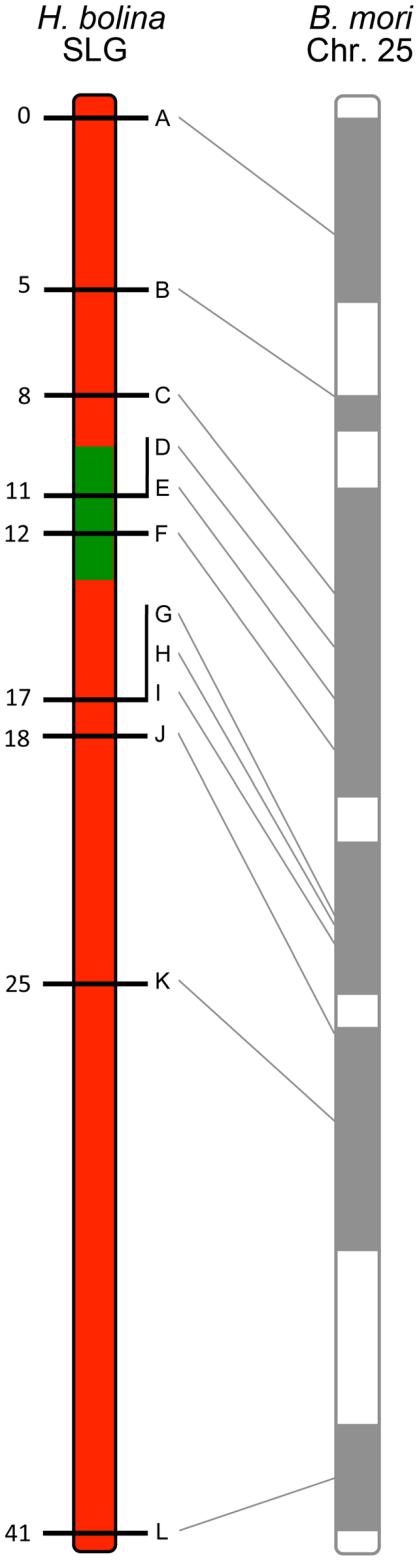
Recombinational map of the *H. bolina* chromosome carrying the suppressor locus. Depiction of the suppressor linkage group (SLG), with the 12 linked markers A-L. The genomic region containing the suppressor locus is highlighted in green. Numbers represent distance from the distal marker in cM as estimated using JoinMap. The broadly syntenic *B. mori* chromosome 25 is given for reference.

A linkage map for this chromosome, the suppressor linkage group (SLG), was then constructed. The region required for male survival was identified by the exclusion of recombinants. This was achieved by examining the segregation of alleles from sons of the SE Asia x Moorea cross above that were mated to *Wolbachia*-infected Moorea (non-suppressor) females (creating a male-informative family). 307 recombinant daughters were obtained, which were used to create a linkage map of the 12 suppressor-linked markers (data used to create linkage map in S6 Table). The markers were estimated to cover a 41 cM recombination distance and were syntenic with *B. mori* (Fig. 1). The suppressor locus was localized to a region within this chromosome by excluding linked loci where the SE Asia derived paternal allele was absent in one or more sons (indicating that the genomic region containing the SE Asia allele was not necessary for male survival). Three suppressor-linked alleles (D, E and F), all in the +11 to +12 region, were retained in all 60 sons, whereas the 9 markers proximal and distal to these were excluded by the presence of one or more recombinants (Fig. 1). The probability of observing retention of a marker in a sample of 60 on the null hypothesis of no association between the +11/+12 genomic region and male survival is 0.5^60^ = 9×10^−19^. Thus we posit that the suppressor lies between marker C at +8 (excluded by one recombinant) and marker G at +17 (excluded by two recombinants) - a region of approximately 10 cM. Our data also indicate that while this genomic region is necessary for male survival, presence of this locus was not always associated with male survival, with the number of surviving sons obtained being one quarter, rather than one half, of the number of daughters obtained in our cross (60 sons *vs* 307 daughters).

### The impact of suppressor spread on linked genetic variation in Samoa

#### a) Modelling a selective sweep driven by suppressor spread

Models of selective sweep dynamics commonly utilize a fixed selective coefficient. In contrast, the intensity of selection is dynamic in our system, as the benefit of rescuing a male relates to population sex ratio, which depends on the frequency of male-killing *Wolbachia* and the frequency of suppression: as the suppressor spreads, the population sex ratio shifts towards 1∶1, thus reducing the selective pressure for further spread. We previously modelled the spread of the suppressor in this system using deterministic calculations [16]. This model tracks genotype frequencies in males and females separately, with suppression encoded at a single dominant locus, as suggested by previous work [6]. For each sex, zygotes are formed by random union of male and female gametes, creating a genotype frequency distribution in zygotes. Male-killing then creates selection between zygotic and adult phases, with any *Wolbachia*-infected males lacking the *S* suppressor gene (i.e. those with genotype *ss*) being killed early in development. Thus the adult pool of male genotypes differs from the zygotic pool, and has a higher frequency of suppression than the adult female pool. Fisherian selection is then implicit in the model, with the suppressor increasing in frequency between the zygote and adult phase by virtue of being overrepresented in male hosts that contribute 50% of the genetic material to the next generation.

In the Samoan population of *H. bolina*, *Wolbachia* was initially at a 99% prevalence in females [5], creating an extremely female biased population sex ratio. Under such circumstances, a novel suppressor mutation will approach 50% frequency in adult males in the first generation (as nearly all surviving F1 males are heterozygous *Ss*), and to 25% frequency in the subsequent generation. Males that survive *Wolbachia* infection by carrying the suppressor are then induced to express another reproductive manipulation commonly employed by *Wolbachia*: cytoplasmic incompatibility (CI) against uninfected females [17]. During CI, the progeny of uninfected females sired by infected males die during embryogenesis, such that the presence of infected males (generated through suppressor action) reduces the fitness of uninfected females relative to infected females (who experience no such reduction in offspring viability following mating with infected males). Despite losing its male-killing ability, the induction of CI through infected males allows *Wolbachia* to remain at or near fixation. As a consequence, selection on the suppressor is also maintained. Indeed fixation for *Wolbachia* in males and females is observed after the spread of the suppressor [11].

**Figure 2:**
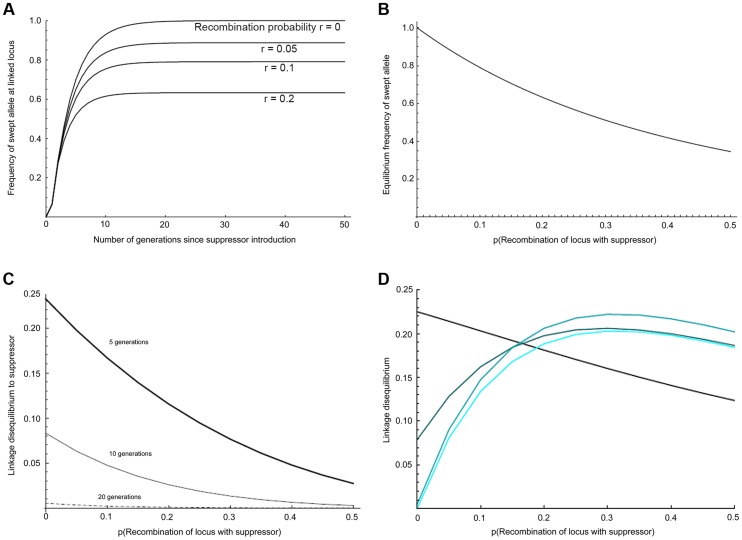
Predicted impact of selective sweep on the frequency and association of linked variants. a) Change in the frequency of initially associated linked variants over time at different recombination distances, b) Equilibrium frequency of initially associated linked variants at different recombination distances, c) Linkage disequilibrium to suppressor for loci at different recombination distance from suppressor after 5, 10 and 20 generations of selection, d) Local linkage disequilibrium between alleles with recombination probability of 0.01 at different recombination distances from the suppressor. The four curves show the situation after 5, 10, 20, and 40 generations (increasing lightness of colour corresponding to greater number of generations).

The expected dynamics of the suppressor locus in this system is given in Fig. 2a, following the trajectory given for *r* = 0 (zero recombination with the suppressor). We elaborated the model of suppressor spread to quantify the expected effects on linked loci under the assumption that the suppressor and linked alleles were cost-free, that recombination occurred in males only, and that spread was occurring through an unstructured, panmictic population (see Supplementary Text S1 for full details). We then modified this model to examine the effect of suppressor spread on levels of association (linkage disequilibrium) between pairs of linked loci, each with two alleles. Within the model, the suppressor locus can have the wild type allele *s*, or the male-killing suppression allele *S*. The second locus is linked to the suppressor locus and has two selectively neutral alternative alleles denoted by *A* and *a*. The model tracks the change in gametic frequencies from one generation to the next. There are therefore four different basic gamete types: *AS*, *As*, *aS*, and *as*. Our individuals are diploid, so these four basic gamete types give nine possible basic individual genotypes: *AASS*, *AaSS*, *aaSS*, *AASs*, *AaSs*, *aaSs*, *AAss*, *Aass*, and *aass*. This is further complicated by the need to record whether individuals are infected or uninfected. Since infection is maternally inherited, we can count infection status as part of the genotype of an individual, giving us a total of eighteen genotypes (the nine above, with each having infected or uninfected status).

Given a set of genotype frequencies for males and females, we can derive the expected gametic frequencies (incorporating recombination in males only), and assuming random mating we can calculate the frequencies of offspring genotypes in the next generation. We can then apply selection: we assume that the infection kills all males lacking the suppressor allele (*ss*), half of the males that are heterozygous (*Ss*) for the suppressor, and none of the males that are homozygous (*SS*) for the suppressor. This partially dominant rescue mirrors the patterns of male survival in our mapping crosses, in which about 60 *Ss* males survive, compared to 150 *Ss* females (estimated survival of male with the suppressor: 40%). For simplicity we assume no other selective effects. This process allows us to track both gametic and genotypic frequencies through time.

We iterated this process for the Samoan situation where 99% of females are infected with a fully penetrant male-killer, in which males only survive in the presence of the suppressor allele, and supposing that the suppressor gene initially appeared in a single infected immigrant male of genotype *AASS*. The selected locus (*r* = 0) is observed to reach over 95% frequency within 10–15 generations, which is consistent with the observed rapid spread of the suppressor (Fig. 2a). Overall, our panmictic model predicts that effects of the sweep are apparent over the entire suppressor-containing chromosome (Fig. 2b), with markers at recombination distance 0.5 to the suppressor locus being swept to 35% frequency. The main cause of this wide impact is selection in the first generation, in which nearly all males in the mating pool are heterozygous for the chromosome carrying the suppressor (thus this chromosome rises to an initial frequency approaching 0.25).

We further examined how the sweep would impact the level of linkage disequilibrium (LD) within the linkage group, both in terms of LD with the suppressor locus (Fig. 2c), and local LD (disequilibrium between pairs of loci other than the suppressor) (Fig. 2d; S1 Text). After 10 generations of selection, association between the suppressor locus and linked loci was weak, and at 20 generations it was expected to be undetectable for a sample of 50 individuals. In contrast, local LD rose and fell over a longer time period. For closely linked loci, LD was expected to be retained even after 40 generations. In addition, local LD was greatest at intermediate recombination distances from the suppressor locus, where swept alleles were at intermediate frequency when at equilibrium.

#### b) Observations of the effect of suppressor spread on patterns of genomic variation between 2001 and 2006

We investigated the change in frequency of genetic variants associated with the spread of suppression of male-killing in *H. bolina* on the island of Upolu, Samoa, through comparison of allelic profiles in the SLG before and after the spread of suppressor (see Methods and Materials). To this end, intronic regions of the 12 suppressor-linked markers (A-L) were sequenced for butterflies from before (2001, n = 48), and after (2006, n = 48) the spread of the suppressor (basic haplotype data and accession numbers given in S2 Table). Haplotype (allele) frequencies at each time point were estimated using PHASE and the results checked manually. Changes in allele frequency distributions at each locus between the pre- and post-sweep samples were examined using a heterogeneity test, and F_ST_ was calculated as a standardized metric of genetic differentiation between population samples. The role of selection in creating the differentiation was examined in three ways. First, we tested whether changes in allele frequency exceed those expected under drift for four marker loci where a novel allele was observed. Second, we investigated the presence of LD between loci in each population sample, the hallmark of recent selection being generation of local LD. Third, evidence of genome-wide changes in allele frequencies was examined, to determine whether the effects seen on the SLG could be associated with general demographic effects. To this end, changes in the frequency of allelic variants were assessed for a control group of 9 loci (M-U) not linked to the suppressor, which derived from 8 different linkage groups.

**Figure 3:**
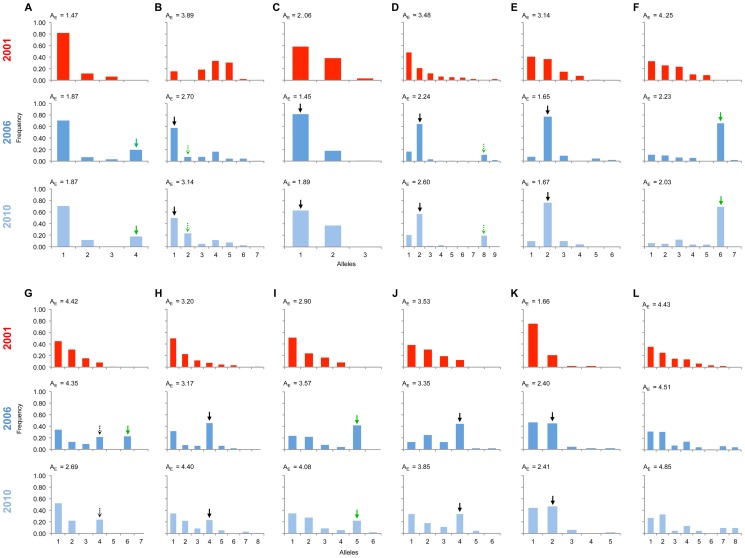
Allele frequency distributions across the linkage group carrying the suppressor in 2001, 2006 & 2010. The major allele variant for each locus (A-L) that has increased in frequency during the sweep is indicated with a solid arrow, while the secondary allele that increases (in 3 cases) is indicated with a dotted arrow. Where the allele is novel in 2006/2010 the arrow is green. Results of statistical comparison presented in S1 Figure. Effective allele numbers (A_E_) are indicated on each graph.

Significant changes in the allele frequency distribution between the 2001 and 2006 population samples were observed at 11 of the 12 markers along the chromosome identified above as carrying the locus necessary for male survival in SE Asian butterflies, spanning a region covering 25 cM (Fig. 3; all 11 with p<0.01 after sequential Bonferroni correction: S2 Figure). Only the most distant marker from the suppressor (marker L) showed no evidence of a difference in allele frequency between 2001 and 2006. An expectation for an episode of selection is that allele frequency distributions will be disturbed by an increase in frequency of any allele initially associated with the target of selection, which would be paralleled by a coordinated decrease in frequency of the other alleles. Because decline is spread across multiple alleles, the expectation is that a swept allele should be the greatest contributor to heterogeneity between allele frequency distributions, and thus identifiable by the largest standardized residual in heterogeneity tests [18]. We performed this analysis of residuals at 10 of the SLG loci where heterogeneity between samples was observed (one locus was not suitable for analysis in this manner, as there were only two allele variants, thus by definition each allele contributes equally to heterogeneity). At each of the 10 loci, a single allele increasing in frequency from 2001 to 2006 contributed the largest residual to heterogeneity analysis, and thus can be regarded as the swept allele. However in 3 cases (loci B, D & G), removal of this allele did not restore homogeneity, and a second allele that increases in frequency can additionally be identified as contributing to the heterogeneity observed, albeit with a lower magnitude of frequency change (swept alleles are annotated with arrows in Fig. 3).

**Figure 4:**
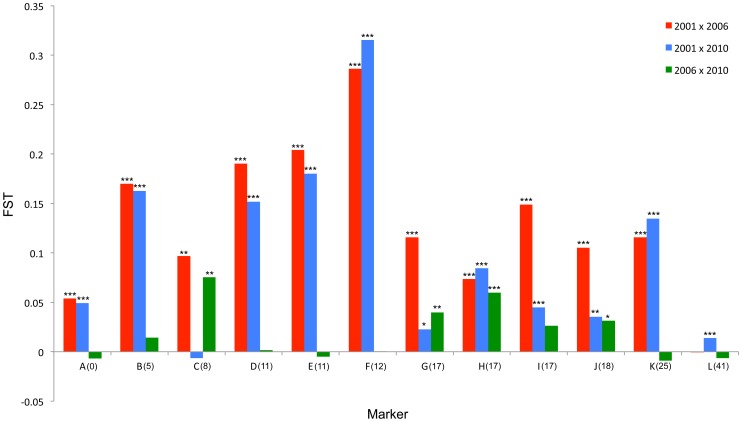
F_ST_ standardized population genetic differentiation between samples from different time points for each locus along the chromosome carrying the suppressor. Blue: 2001–2006; Red: 2001–2010; Green: 2006–2010. Statistically significant deviations from F_ST_ = 0 given by *** (p<0.001), ** (p<0.01) * (p<0.05) uncorrected for multiple tests. Depiction of suppressor linkage group given for reference. Position along SLG given in parenthesis on X-axis.

We calculated the F_ST_ statistic for each locus in the SLG as a standardized magnitude of differentiation between the 2001 and 2006 population samples (Fig. 4), and A_E_, the effective number of alleles present in the population, as a measure of overall diversity (shown in Fig. 3 above graphs). F_ST_ was highest in the 5 cM region in which the suppressor had been previously located in SE Asian butterflies (loci D–F, F_ST_ = 0.2–0.3), and in this region allele diversity (A_E_) approximately halved between 2001 and 2006. Lower, but significant, F_ST_ was found over a 25 cM region surrounding the locus (p<0.01 at all loci after sequential Bonferroni correction), but there was no overall change in allele diversity at loci situated further from the suppressor locus. Changes in nucleotide diversity between 2001 and 2006 echoed changes in allele diversity. Reductions in π were observed at the three loci that co-segregated with suppression (D–F), while there was little or no perturbation in overall diversity outside this region (S3 Table). The observation that the greatest magnitude of change occurs in loci co-segregating with suppression is consistent with an episode of selection focused within this region.

Further evidence of the role of selection in driving the change in allele frequency on chromosome 25 derives from three sources: a) the magnitude of changes observed compared to those expected from drift processes, b) generation of LD in the 2006 post-suppressor spread population, and c) lack of significant changes in unlinked loci. The magnitude of changes expected under drift can be ascertained from the variance of a neutral Wright-Fisher process. The expected variance in allele frequency after *t* generations for alleles that start with frequency *p* in a population of size *N* is *p*(1−*p*)(1−exp[−*t*/2*N*]), with the standard deviation given by the square root of this measure. We use this formulation to examine the magnitude of change accountable for by drift between 2001 and 2006. We do this for the simplest cases, loci A, F, G and I, which cover 17 cM surrounding the suppressor locus, and are characterized by novel alleles in the 2006 sample. We make the conservative assumption that each allele is at the upper 95% confidence interval for its starting frequency, 0.0377, in 2001, and that there are 10 generations per year. Putting *t* = 50, *p* = 0.0377 and *N* = 500, it is improbable that change associated with drift between 2001 and 2006, could cause changes in allele frequency greater than 3 standard deviations, or 0.12. In each case, the alleles that have entered the population have attained frequency in excess of 0.2, suggesting that drift alone cannot explain the observed changes.

**Figure 5:**
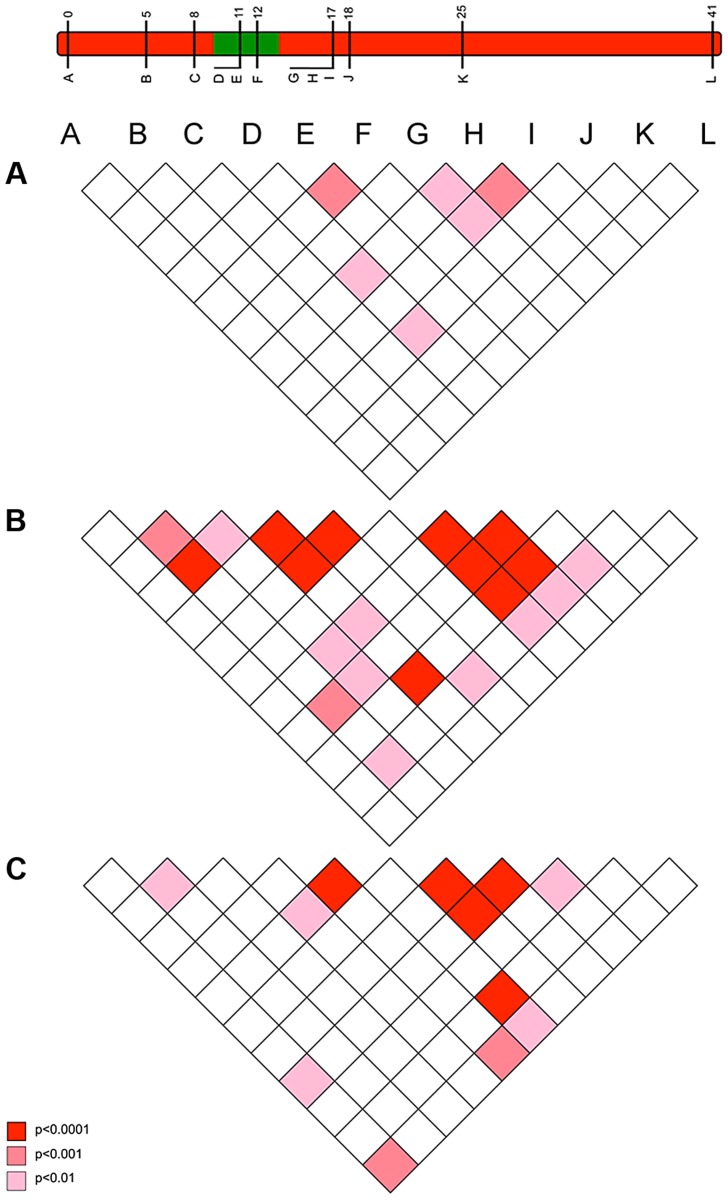
The pattern of linkage disequilibrium (association) between loci observed in 2001 (pre-sweep), 2006 (immediately post-sweep) & 2010 (4 years post sweep) population samples. Pattern of linkage disequilibrium (LD) between suppressor-linked loci (A-L) for a) 2001 (pre-sweep), b) 2006 (immediately post-sweep), and c) 2010 (4 years post-sweep). Significance denoted by colour: deep red – significant LD as measured at p<0.001; mid red – significant LD as measured at p<0.01; pink – significant LD as measured at p<0.05, all uncorrected for multiple tests. Depiction of suppressor linkage group given for reference.

The pattern of LD also reflected an episode of selection in this genomic region. Our model above predicted that LD between the suppressor-linked loci and the target of selection (global LD) would exist only in the very early phases of the sweep (10–20 generations), but that the sweep would create multiple local associations between closely linked loci that would be retained over longer periods (local LD) (Figs. 2c and 2d). In accord with this, there was little evidence of LD between loci in the 2001 pre-suppressor sweep sample, but strong associations between variants at closely linked loci (r = 0.01–0.02) in the 2006 sample (Fig. 5).

Final evidence for the role of selection in driving allele frequency distribution changes on this chromosome derive from the lack of change in unlinked markers. In contrast to the data from the SLG, none of the 9 unlinked markers show evidence of a change in allele frequency between 2001 and 2006 (NS at p = 0.05 after sequential Bonferroni correction; S3 Figure), and little or no change in nucleotide diversity, π (S4 Table). Furthermore, F_ST_ was either zero or very low (F_ST_<0.015) in this group of markers (S5 Table). The lack of change in the allele frequency distribution at unlinked loci further allows us to reject demographic factors and drift as causes of changes in allele frequencies in the suppressor linkage group.

#### c) Observations of the pattern of genetic variation at suppressor-linked loci post 2006

In order to investigate the long-term impact of the spread of the suppressor, and identify whether this episode of selection was near complete in 2006, we also analysed the pattern of variation within the linkage group for a further population sample (n = 46) collected in 2010 (*c.* 32 generations after the 2006 sample). Local LD was still observed, but, as expected from continued recombinational erosion, the extent of LD was confined to very tightly linked loci (Fig. 5c). In terms of the allelic profile, the pattern observed was heterogeneous (Fig. 3; Fig. 4). At six loci (including the three in the direct vicinity of the region containing the suppressor), allele frequencies in the 2010 sample were not significantly different from the 2006 sample. Differentiation between the 2001 and 2010 samples, as measured by F_ST_, was approximately equivalent to that previously observed in 2006. The allele frequency distributions at these loci are apparently at equilibrium, and from this we conclude that they were no longer being selected by association with the suppressor. However, allele frequency distributions differed between the 2006 and 2010 samples at the remaining 5 loci located across two genomic regions, one proximal to that carrying the suppressor, and one distal to it. For these, differentiation between the 2001 and 2010 samples was also reduced, and significant differentiation of the 2010 to the 2006 sample was also observed (Fig. 4). In all cases the differentiation is caused by the loss (or reduction in frequency) of the major allele that had been swept between 2001 and 2006. Our sampling was geographically broad for both 2006 and 2010 samples, leading us to reject population substructure as a cause of allele frequency heterogeneity. A last feature to note in the 2010 sample is that locus L, the most distal locus from the suppressor, did show evidence of heterogeneity between 2001 and 2010, in contrast to the lack of heterogeneity between 2001 and 2006 samples. While it would be premature to conclude that this locus has also been subject to selection through linkage with the suppressor (as the quantity of change is within that which drift could achieve), this observation leads us to conclude that changes in the pattern of allele variation may actually extend throughout the length of this linkage group.

We finally examined whether the loss of originally swept suppressor-linked alleles by 2010 could be explained by genetic drift. Using the model of Kimura ([19], equation 7), we ascertained the effective population size at which there is a reasonable chance that an allele present in 2006 would be absent in 2010. We conducted this test for locus G, where the swept allele was at a frequency of 0.22 (n = 94) in 2006 and absent in 2010. Setting the time elapsed between samples conservatively at 40 generations (4 years, 10 generations per year based on an egg-mature adult period of 36 days), we estimated that loss would occur on 1% of occasions with *N_e_* = 180, and on 5% of occasions with *N_e_* = 122. Given the size of Upolu, Samoa, and the ease of collecting adult butterflies (our samples of 48 were collected in two days from three sampling points, and did not involve sampling pupae/larvae), we consider it likely that *N_e_* is considerably larger than this, and thus conclude that drift was unlikely to cause the loss of material observed. Furthermore, we note that loss of introgressed material occurred at two genomic locations that were not in LD with each other, again making chance sampling an unlikely source of the loss of material. Rather, we hypothesize that purifying selection against introgressed material is the most likely reason for the loss of swept alleles.

## Discussion

Our data identified a 10 cM genomic region on chromosome 25 of SE Asian *H. bolina* that was necessary for a male butterfly to survive *Wolbachia* induced male-killing. This region was also a focus of selection during the spread of suppression of male-killing between 2001 and 2006 in Samoa. During this episode, patterns of allelic variation were observed to be altered over a 25 cM region of chromosome 25, with increases in frequency of one allele at each locus creating the vast majority of heterogeneity between time points. The largest magnitude of change occurred in markers that co-segregated with suppression in SE Asia, and in this region the overall genetic diversity (as measured by A_E_ and π) declined - the classical signature of a selective sweep. Three further features implicate the role of selection in altering allele frequency across this 25 cM region. First, the changes in allele frequency are too large to be accounted for by drift, even under conservative assumptions for population size and generation time. Second, LD is generated across this region, as predicted under a model of strong selection. Third, 9 markers unlinked to the suppressor linkage group showed no evidence of changes in the frequency of allele variants between 2001 and 2006, implying that demographic factors were not the major force driving changes in allele frequency.

While we observed changes consistent with the operation of selection over a very broad genomic area, the degree of change was less than that predicted from our model. This is true both of the magnitude of allele frequency change at loci located near the suppressor locus, and the breadth of the region of chromosome over which changes in allele frequency occurred. Our model, which presumes a panmictic model and no cost to carrying the suppressor, predicts the suppressor should fix (and take alleles within 5 cM distance to frequency in excess of 87%), and that allele frequency changes should be observed chromosome-wide. In contrast, the swept allele at locus D (which lies within 5 cM of the target of selection) attains a frequency of just 0.67 (n = 172, CI 0.59–0.74) in 2006 and 2010 samples. Further, we observed only very small changes in allele frequency at the most distant locus from the region containing the suppressor, locus L.

We suggest there are three non-mutually exclusive explanations for this lack of fit with the model. First, the suppressor mutation in natural populations diffuses spatially following its initial arrival, and each generation of spatial diffusion is associated with a narrower local sweep. The principle impact of spatial diffusion will be to narrow the genomic region that is affected by selection compared to that predicted in a panmictic model, and to reduce the magnitude of change at loci far from the target of selection. For a locus 25 cM distant from the suppressor, association with the suppressor allele may last just one or two generations, such that changes in allele frequency occur only near the point of origin, and are diluted by absence of any selection on these loci in the majority of the species range. However, spatial diffusion represents a poor explanation for the lower than expected frequency of variants at tightly linked loci post-sweep, which are expected to maintain strong association during the spread of the suppressor across the island, as this occurs in about 10 generations. A second possibility is the involvement of other loci in the genome, as enhancers of suppressor action. Our data indicate that the genomic region in question is necessary for male survival, but do not rule out involvement of other loci in enhancing suppression. If other loci are involved, either as required elements or enhancers of male survival, this would slow suppressor spread, and might account for the narrowness of the sweep observed compared to model predictions. However, the requirement of the genomic region for male survival in the presence of *Wolbachia* again makes this a poor explanation for the failure of tightly linked loci to reach high frequency. Because it is necessary, it should become fixed in the population, and closely associated allele variants should in consequence attain very high frequency. A third possibility is that there is a cost to being homozygous for the suppressor mutation, either in both sexes, or in female hosts only. A cost such as this could prevent fixation of the suppressor allele, and thus also help account for the decreased magnitude of effect at loci tightly linked to the suppressor. If the suppressor allele reaches >0.75 frequency, then males lacking the suppressor would be sufficiently rare that the population sex ratio would be near parity. Biologically, costs to suppressor carriage may be directly associated with the suppression system itself. Modification of a sex determination gene, for instance, might rescue males but be deleterious in females, or when homozygous. Alternatively, costs may be associated with linked mutations. The presence of deleterious loci in linkage with the suppressor is supported by our observation that some material that had been initially swept into the population was lost between 2006 and 2010. Finer-scale investigation of this linkage group, especially within the region identified as required for male survival, is necessary to illuminate the precise dynamics that occurred during this episode of selection.

In our data, we observed concordance between the position of the suppressor ascertained in SE Asian butterflies, and the genomic region subject to selection during spread of suppression through the Samoan population of the butterfly. This observation has two possible interpretations. First, the suppressor mutation may have been introduced into Samoa by migration. Given that the suppressor is absent in the nearest island groups, American Samoa and Fiji, suppressor introduction would be associated with a long distant migrant. Second, the genomic region identified here may represent a hotspot for suppressor mutation, derived independently in Samoa by *de novo* mutation. This may be an identical mutation to that found in SE Asia, or an alternative mutation in the same gene, which still confers suppression. Alternatively, there may be a suppression-conferring mutation in a different gene within the region identified as containing the suppressor.

The presence of novel swept alleles at loci linked to the suppressor indicates that migration is the most parsimonious explanation for suppressor origin. Variants not present in the 2001 sample were observed to be the main ‘swept’ allele at 4 of the 11 loci at which significant change was detected (indicated with green arrows in Fig. 3). At two of these loci (A & I), the invading allele was defined by a single nucleotide polymorphism (SNP) being absent from the 2001 sample, whereas the other two alleles represented different combinations of existing SNPs. The four loci were in three genomic locations spaced over 17 cM and showed no evidence of linkage disequilbrium in the 2001 pre-sweep sample, and thus they can be treated as independent from each other (Fig. 5). They therefore support (but do not definitely prove) a migratory origin.

None of the loci tested in this study are likely to be the suppressor locus itself (markers were selected that spanned chromosome 25 and had conserved exon sequence – with several being housekeeping genes). Future research should aim to establish the actual nature of the suppressor mutation in both Samoa and SE Asia through fine-scale genetic mapping. Such a project will allow the source of suppression on Samoa (migration or in situ mutation) to be clarified. Beyond this, it will reveal the actual target of selection in this system. It has been widely conjectured that the evolution of sex determination systems might occur in response to the presence of sex ratio distorting microbes [20]. It is notable that a strong candidate gene – *doublesex* – resides within the equivalent genomic area in *Bombyx mori*, and with conservation of synteny being profound in Lepidoptera, is likely to reside in this area in *Hypolimnas*. *Doublesex* represents a tempting candidate as it is known that splicing of this gene is altered in the presence of male-killing *Wolbachia* in another lepidopteran, the moth *Ostrinia scapulalis* [21].

## Materials and Methods

### Developing a genome-wide marker set for *H. bolina*

We utilized high-throughput sequencing of the transcriptome of *H. bolina* to obtain coding sequence from multiple loci across the genome. Following total RNA extraction from 1 male and 1 female adult *H. bolina*, mRNA library construction and sequencing using the Roche 454 sequencing platform (http://www.454.com), 450 bp reads were *de novo* assembled into contigs using the Newbler assembler to create the first set of Expressed Sequence Tags (EST) for *H. bolina*. The trimmed reads have been deposited as one male, and one female, partial transcriptome datasets in the NCBI SRA database, accession SRP045735.

In the absence of any annotated genome or transcriptome for *H. bolina*, the moth *Bombyx mori* was used as a proxy reference genome, this being the only available resource for Lepidoptera at the time of the study. There is a high level of synteny of gene location in the Lepidoptera [22] allowing a targeted gene approach, in which several genes could be selected from each chromosome across the genome. Coding sequence of highly conserved genes such as ribosomal proteins and housekeeping genes from *B. mori* were initially targeted and then retrieved from NCBI (http://www.ncbi.nlm.nih.gov). To determine putative *H. bolina* orthologs a local tBLASTx was then performed against the *H. bolina* EST set. Only genes that returned a single tBLASTx hit were included, reducing the likelihood of the inclusion of paralogs in our marker set. The orthologous *H. bolina* contigs were then translated into amino acid sequences using the ExPASY online tool (http://web.expasy.org/translate), with the sequence lacking mid-sequence stop codons chosen as the most likely translation. In a final test for paralogs, a reciprocal BLAST was performed of coding sequence from the orthologous *H. bolina* contigs as queries against the *B. mori* genome using the INPARANOID8 search tool ([23]; http://inparanoid.sbc.su.se/).

Where present, intronic regions were targeted for marker development, as they are likely to have a higher degree of nucleotide diversity. Again, conservation of synteny in Lepidoptera genome organisation allowed the intron/exon boundaries in *H. bolina* genes to be inferred using the *B. mori* genome. Through tBLASTx analysis of the *B. mori* coding sequence of the targeted gene against the *B. mori* WGS (Whole Genome Shotgun contigs) database in NCBI, exonic regions were identified (as only these regions will align). The translated orthologous *H. bolina* contig and the corresponding *B. mori* amino acid sequence were aligned using ClustalW [24] and the position of the intron/exon boundaries subsequently located.

Once intron/exon boundaries were identified in *B. mori* genes, and extrapolated to the *H. bolina* orthologous sequences, primers were designed for *H. bolina* that spanned introns of size 500–1000 bp (*Bombyx* size approximation). This size range was chosen to enable successful amplification of the intronic region during PCR. Marker optimisation was performed using three test *H. bolina* samples and successful PCR products were sequenced using Sanger technology.

### Mapping the suppressor linkage group

In order to investigate the genetic architecture of male-killing suppression in *H. bolina* and determine markers in linkage with the suppressor locus, we crossed females of a butterfly population (the Philippines) that were *Wolbachia*-infected and homozygous for the male-killing suppressor allele (***SS***) to males from a *Wolbachia*-infected population (Moorea, French Polynesia) that lacked the suppressor (*ss*), to create suppressor-heterozygous *Wolbachia*-infected offspring (***S*** *s*) (S1 Figure).

Recombination does not occur during female meiosis in the Lepidoptera [25], permitting the progeny of ***S*** *s* females to be used to identify the linkage group (SLG, Suppressor Linkage group) in which the dominant suppressor allele was carried. To this end, ***S*** *s* females were crossed with *ss* males to produce the female-informative families. For inclusion in the SLG, markers linked to the suppressor locus are characterized by being present in all surviving sons of the ***S*** *s* heterozygous mother, rather than the 50% expectation from Mendelian segregation with random survival. Initially each marker was sequenced in the F1 parents (***S*** *s* female×*ss* male). In each case, SNPs were chosen that were heterozygous in the female and homozygous in the male – following the presumed pattern of the suppressor. These same SNPs were then scored in 16 male and 8 female F2 progeny. Once a marker had been found that was present in half of the daughters (following Mendelian inheritance) but all of the sons (for a son to survive it must have at least one copy of the suppressor, and hence linked marker allele), further markers were developed for that same chromosome based on synteny with *B. mori*. A final suite of 12 markers that produced clean sequence and that spanned the suppressor-associated chromosome were developed to form the SLG.

Recombination does occur in male *H. bolina*, and thus crosses of ***S*** *s* males to *ss* females (the male-informative families) allow a) mapping of genetic markers within a chromosome relative to each other and b) mapping of the suppressor within the linkage group, in terms of a region of the chromosome that is always present in surviving sons. To this end, the 12 linked markers were sequenced in the female F2 (n = 307) from one male informative cross (***S*** *s* male×*ss* female) and a linkage map created using JoinMap (version 3.0; Haldane mapping function) [26]. To place the suppressor locus within the map F2 males (n = 60) from this cross were analysed using the same 12 markers. Absence of recombinants in a core subset of markers, flanked by markers with an increasing numbers of recombinants, indicated the position of the suppressor locus (Fig. 1).

### Assessing the effect of suppressor spread on genomic variation

A population sample of butterflies from three time points (2001: n = 48, 2006: n = 48, 2010: n = 46) were collected from the Samoan island of Upolu. For each individual, DNA was extracted using the Qiagen DNeasy kit (www.qiagen.com), and the suite of 12 suppressor-linked markers amplified using PCR. Following Sanger sequencing of the amplicons through both strands, the resultant marker sequences were alignment in Codoncode (www.codoncode.com/). SNPs present within and between the population samples were then identified and scored for each individual butterfly. Using the SNP data (given in S7 Table), the alleles present at each marker in each population sample were estimated using the haplotype reconstruction software PHASE (version 2.1 [27], [28]) with 1000 iterations, a thinning interval of 100 and 1000 burn-in iterations. Allele frequencies at each marker for each time group could then be calculated and compared. Output was also examined by eye, with alleles identified first where there was no ambiguity (either homozygous, or a SNP separating into two defined alleles). Thereafter, alleles were assumed identical to those already identified where possible. The low allele diversity meant this visual analysis produced very similar result to PHASE output, which can thus be considered robust.

Patterns of genetic differentiation were estimated using GENEPOP [29]. First, heterogeneity of allele frequency distributions between pairs of time points was estimated using a G test based on allele frequency distribution. Where allele distributions were heterogeneous, we ascertained the allele whose frequency change made the greatest contribution to heterogeneity as that with the largest standardized residual within the heterogeneity test [18]. This allele was then removed (it was an allele increasing in frequency in each case), and the data retested to ascertain if the population samples were then homogeneous, or whether there was evidence for a second allele that changed in frequency (a second allele was identified in three cases). We additionally used F_ST_ standardized population genetic differentiation to quantify the magnitude of change between allele frequency distributions between the two samples. In each case, the rare individuals where sequence could not be obtained for particular alleles, or not inferred accurately, were coded as missing information.

DNA polymorphism statistics and estimates of nucleotide diversity (number of segregating sites, number of haplotypes, pi, theta, the average number of nucleotide sequences (*k*), Tajima's D, haplotype diversity (*Hd*)) for each marker for each time point were conducted in DnaSP (version 5) [30]. These statistics were estimated using sequence data excluding gaps i.e. indel mutations were not used (present in 8 of 9 unlinked markers).

Nine unlinked markers, from 8 different chromosomes, were also sequenced for the 2001 and 2006 population samples to investigate the degree to which changes were observed in the wider genome and as a control for demographic effects. These were tested for the presence of heterogeneity between time points using a G test based on allele frequency distributions, for differentiation using the F_ST_ statistic, and several polymorphism statistics as described above for the SLG markers.

We additionally analysed evidence for alteration in the pattern of linkage disequilibrium, again using GENEPOP. The significance of LD between all possible combinations of loci was tested in the 2001 and 2006 samples separately. We do not report the magnitude of LD, as this is not a standardized measure, being dependent on the allele frequency distribution at each locus.

## Supporting Information

S1 Figure **Mapping of the** ***Hypolimnas bolina*** **genomic region surrounding the suppressor of male-killing.** A *Wolbachia* infected (denoted by ‘w’) female that was homozygous for the suppressor allele (***SS***) was crossed to a uninfected male that did not carry this allele (*ss*). To produce a female-informative family, *Wolbachia*-infected heterozygous daughters (***S*** *s*) from this pairing were in turn crossed to uninfected males lacking the suppressor. Because there is no recombination in female Lepidoptera, male survival is associated with inheritance of the linkage group carrying the suppressor, and suppressor-linked loci can be identified as those present in all surviving F2 sons (those marked with a cross die) but only 50% of F2 daughters. To produce a male-informative family, *Wolbachia*-infected heterozygous sons (***S*** *s*) from the original parental cross were crossed to infected females lacking the suppressor. Using this cross, members of the suppressor-associated linkage group were mapped relative to each other through the pattern of recombination in the F2 daughters. The location of the suppressor was ascertained as the genomic region that was present in all surviving F2 sons.
(TIF)

S2 Figure **Results of statistical testing for genotypic differentiation between population samples at loci in the linkage group carrying the suppressor.** Significance denoted by colour: deep red – significant differentiation as measured at p<0.001; mid red – significant differentiation as measured at p<0.01; pink – significant differentiation as measured at p<0.05, all uncorrected for multiple tests.
(TIF)

S3 Figure **Allele frequency profiles of markers unlinked to the suppressor.** Allele frequency changes for all 9 unlinked alleles (M-U) between 2001 (red) and 2006 (blue) at all 9 loci were not significant (Chi Square heterogeneity test; p>0.05, Bonferroni corrected).
(TIF)

S1 Table **Marker loci information.** Information of each of the 21 (12 suppressor-linked, and 9 unlinked) loci used in this study including gene annotation, linkage group and primers used. SLG: Suppressor Linkage Group; nt: nucleotides.
(TIF)

S2 Table **Basic haplotype polymorphism information and accession numbers.**
(TIF)

S3 Table **Polymorphism statistics and nucleotide diversity estimates for the 12 suppressor-linked loci (A-L) for the 2001, 2006 and 2010 Samoan population samples.** #: number.
(TIF)

S4 Table **Polymorphism statistics and nucleotide diversity estimates for the 9 unlinked loci (M-U) for the 2001 and 2006 Samoan population samples.** Only SNPs were used in these analyses, indels where excluded. **#**: number.
(TIF)

S5 Table **F_ST_ analysis of differentiation between 2001 and 2006 Samoan population samples at the nine unlinked loci.**
(TIF)

S6 Table **Data generated for linkage mapping.** Data for 307 daughters of the male-informative cross in the format required for Joinmap.
(XLSX)

S7 Table **Genotypes for each individual used in the study, for the 12 suppressor-linked markers.** Each SNP is denoted using the IUPAC code. Missing data occurred where the sequence was not scorable for that individual.
(XLSX)

S1 Text **Modelling the spread of the suppressor.**
(DOCX)
