# Supplementary figures and images for: The Evolution of Sex Ratio Distorter Suppression Affects a 25 cM Genomic Region in the Butterfly Hypolimnas bolina
Source: PLoS Genet. 2014 Dec 4;10(12):e1004822. doi: 10.1371/journal.pgen.1004822 (PMC4256269; doi:10.1371/journal.pgen.1004822)

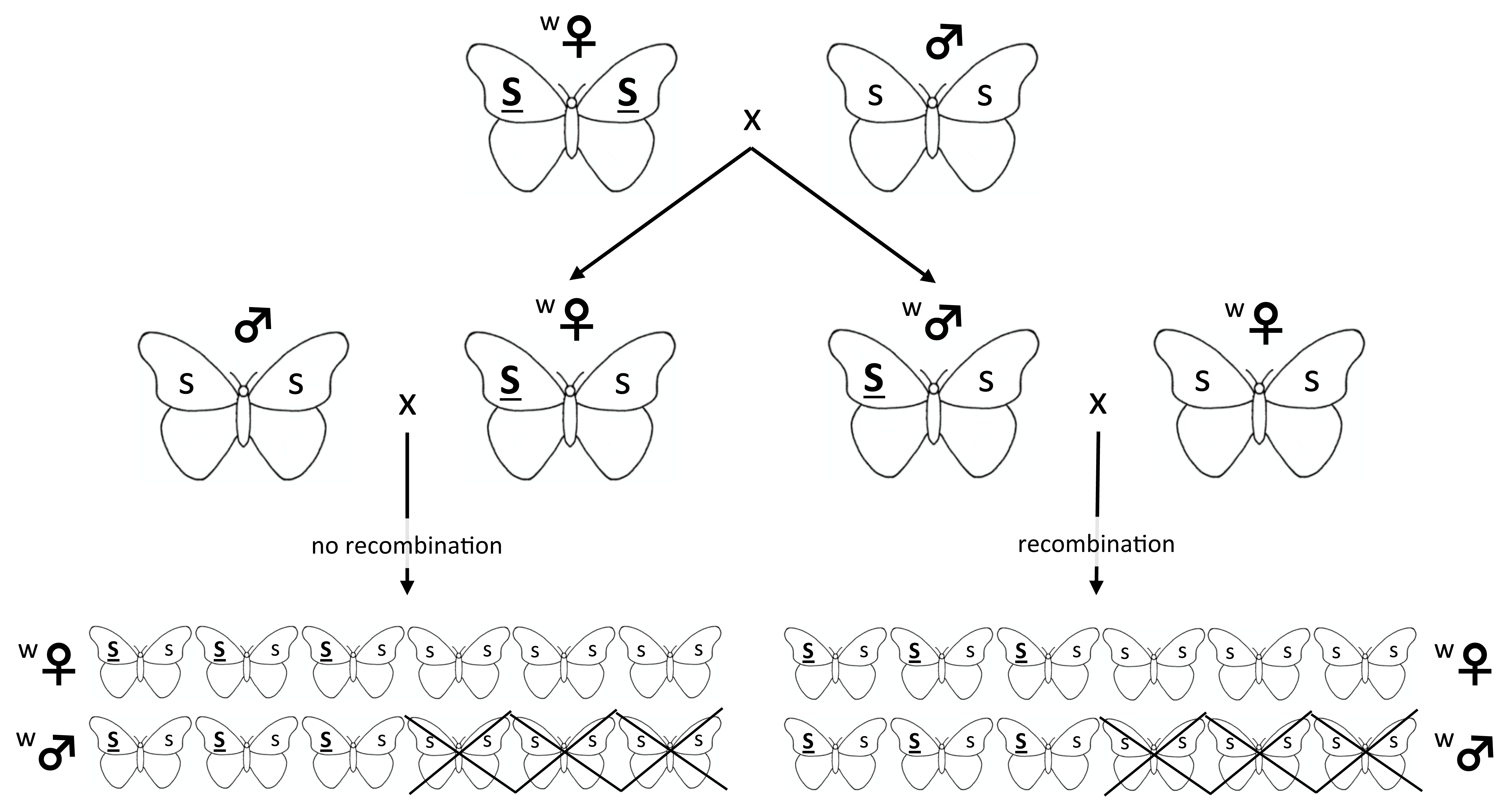

Supplement: S1 Figure — Mapping of the Hypolimnas bolina genomic region surrounding the suppressor of male-killing. A Wolbachia infected (denoted by ‘w’) female that was homozygous for the suppressor allele (SS) was crossed to a uninfected male that did not carry this allele (ss). To produce a female-informative family, Wolbachia-infected heterozygous daughters (S s) from this pairing were in turn crossed to uninfected males lacking the suppressor. Because there is no recombination in female Lepidoptera, male survival is associated with inheritance of the linkage group carrying the suppressor, and suppressor-linked loci can be identified as those present in all surviving F2 sons (those marked with a cross die) but only 50% of F2 daughters. To produce a male-informative family, Wolbachia-infected heterozygous sons (S s) from the original parental cross were crossed to infected females lacking the suppressor. Using this cross, members of the suppressor-associated linkage group were mapped relative to each other through the pattern of recombination in the F2 daughters. The location of the suppressor was ascertained as the genomic region that was present in all surviving F2 sons. (TIF) [file pgen.1004822.s001.tif]

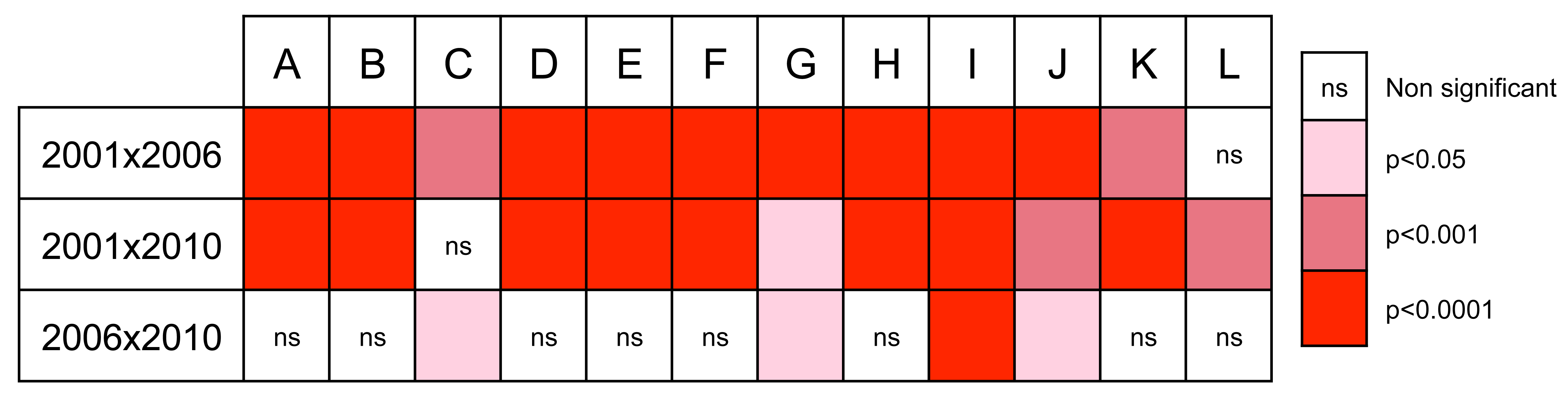

Supplement: S2 Figure — Results of statistical testing for genotypic differentiation between population samples at loci in the linkage group carrying the suppressor. Significance denoted by colour: deep red – significant differentiation as measured at p<0.001; mid red – significant differentiation as measured at p<0.01; pink – significant differentiation as measured at p<0.05, all uncorrected for multiple tests. (TIF) [file pgen.1004822.s002.tif]

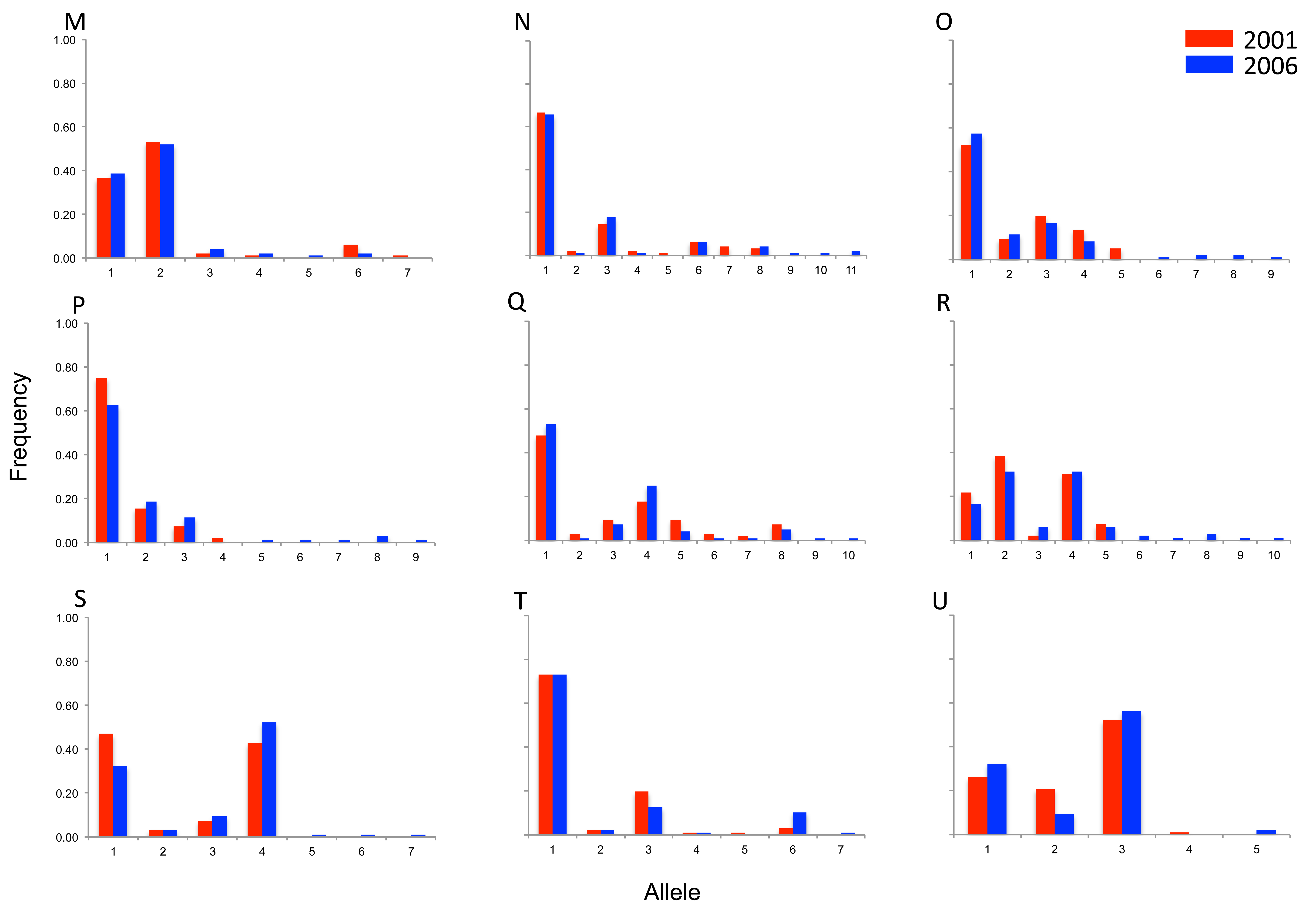

Supplement: S3 Figure — Allele frequency profiles of markers unlinked to the suppressor. Allele frequency changes for all 9 unlinked alleles (M-U) between 2001 (red) and 2006 (blue) at all 9 loci were not significant (Chi Square heterogeneity test; p>0.05, Bonferroni corrected). (TIF) [file pgen.1004822.s003.tif]

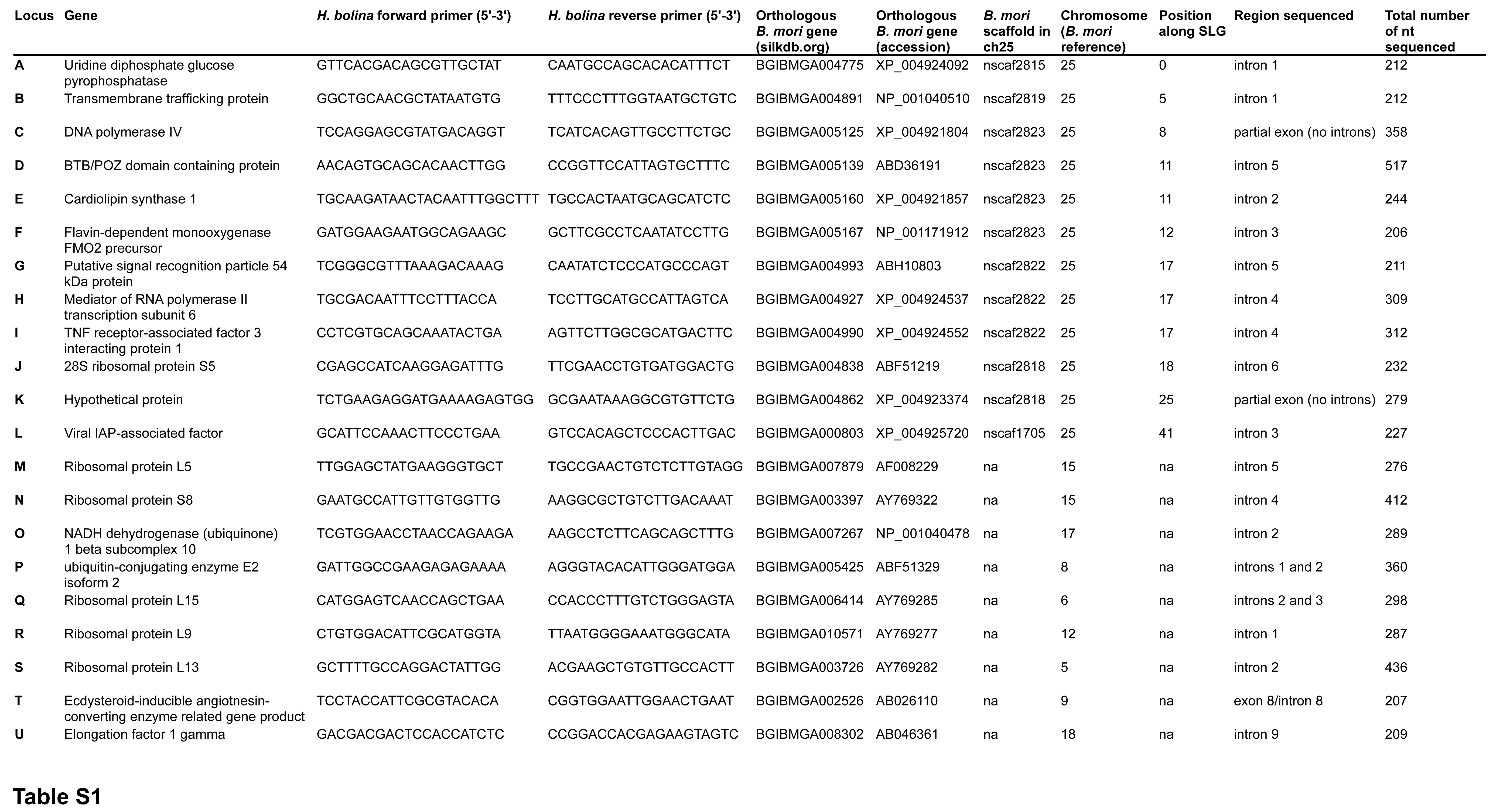

Supplement: S1 Table — Marker loci information. Information of each of the 21 (12 suppressor-linked, and 9 unlinked) loci used in this study including gene annotation, linkage group and primers used. SLG: Suppressor Linkage Group; nt: nucleotides. (TIF) [file pgen.1004822.s004.tif]

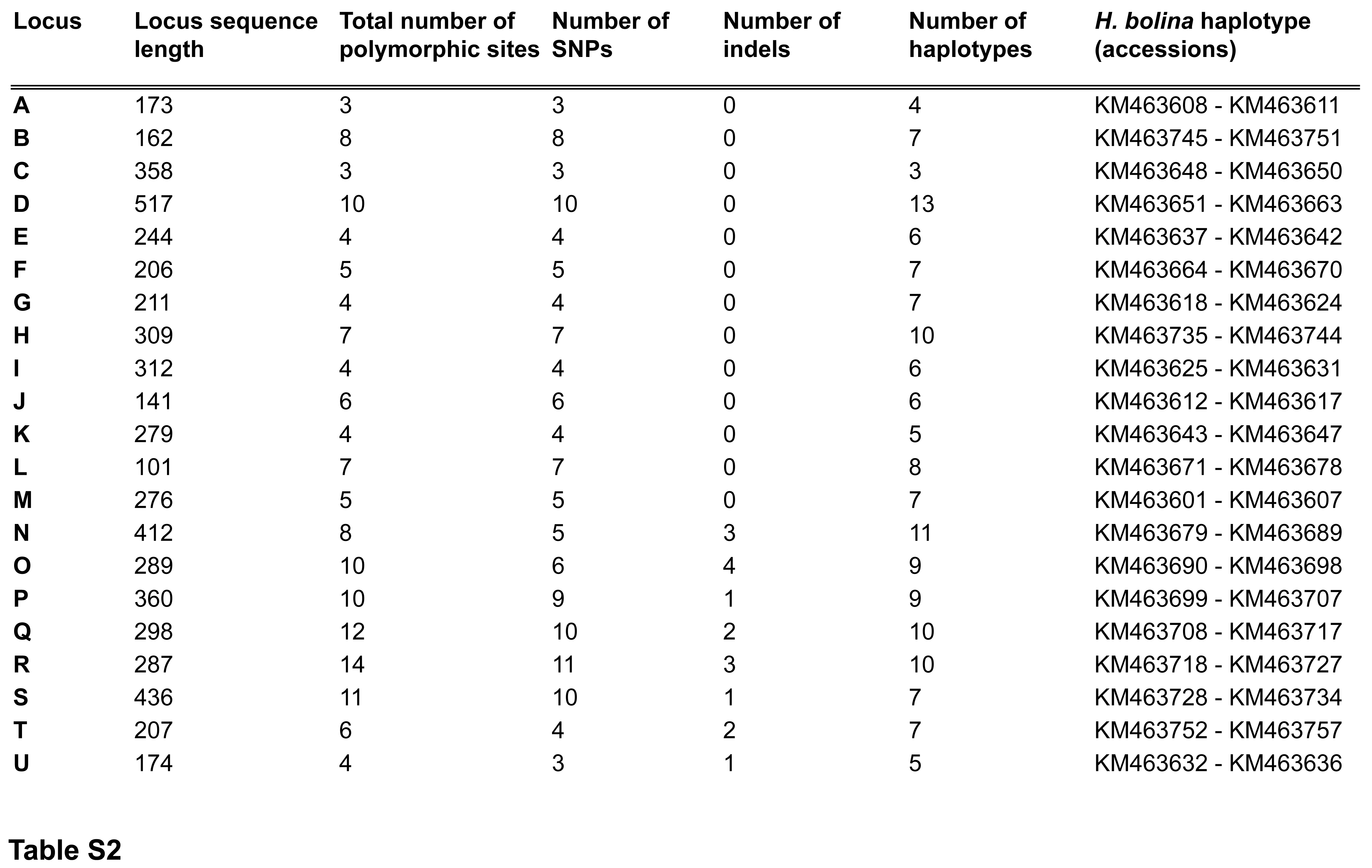

Supplement: S2 Table — Basic haplotype polymorphism information and accession numbers. (TIF) [file pgen.1004822.s005.tif]

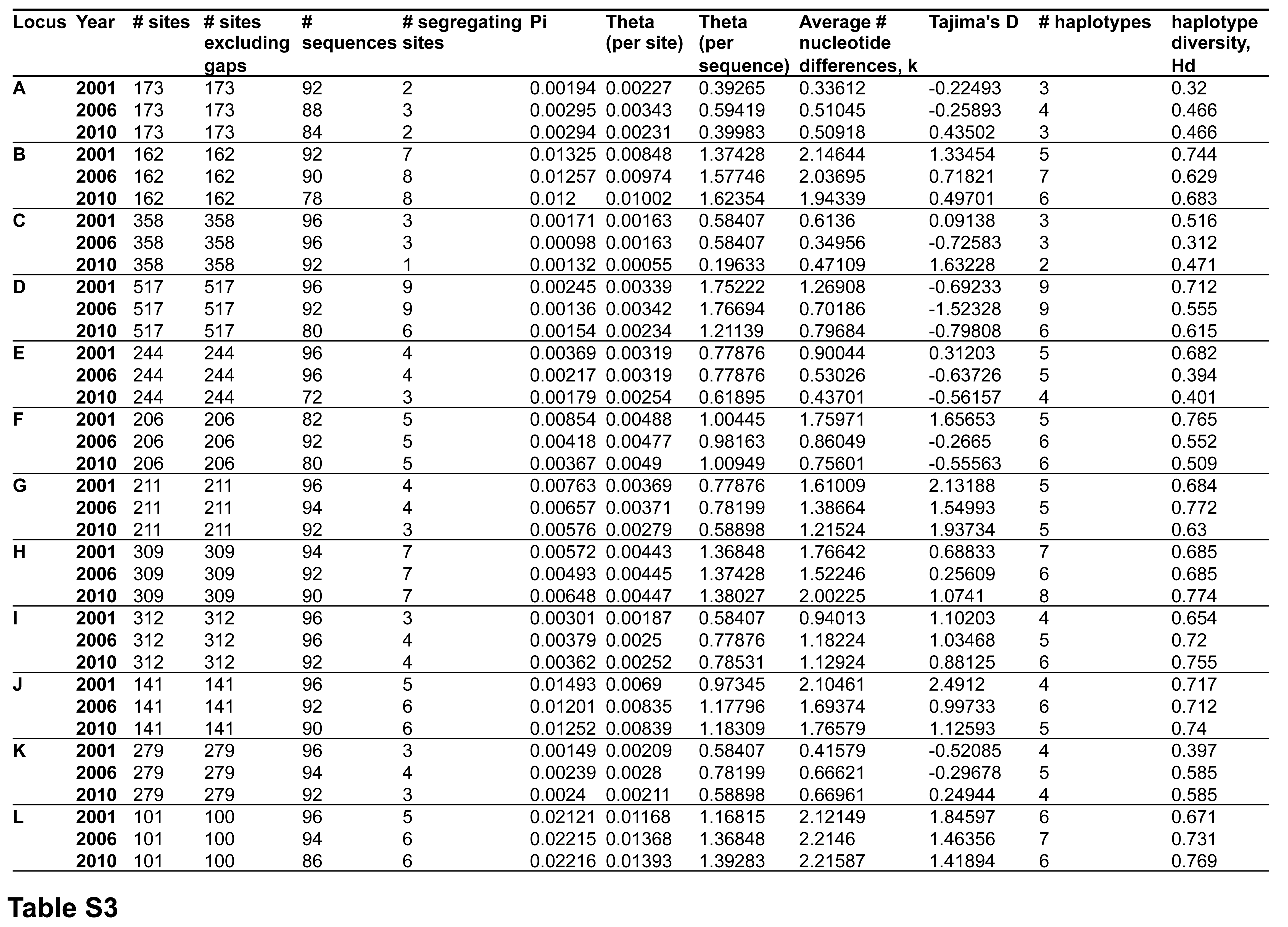

Supplement: S3 Table — Polymorphism statistics and nucleotide diversity estimates for the 12 suppressor-linked loci (A-L) for the 2001, 2006 and 2010 Samoan population samples. #: number. (TIF) [file pgen.1004822.s006.tif]

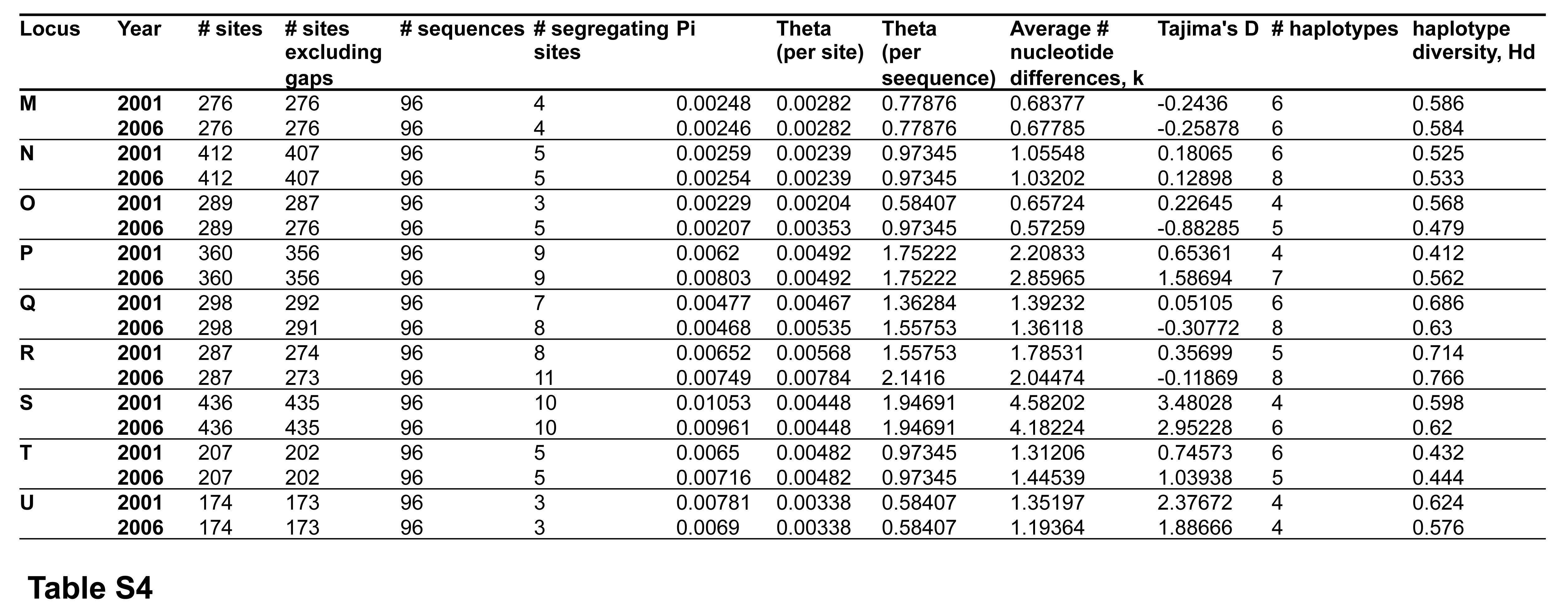

Supplement: S4 Table — Polymorphism statistics and nucleotide diversity estimates for the 9 unlinked loci (M-U) for the 2001 and 2006 Samoan population samples. Only SNPs were used in these analyses, indels where excluded. #: number. (TIF) [file pgen.1004822.s007.tif]

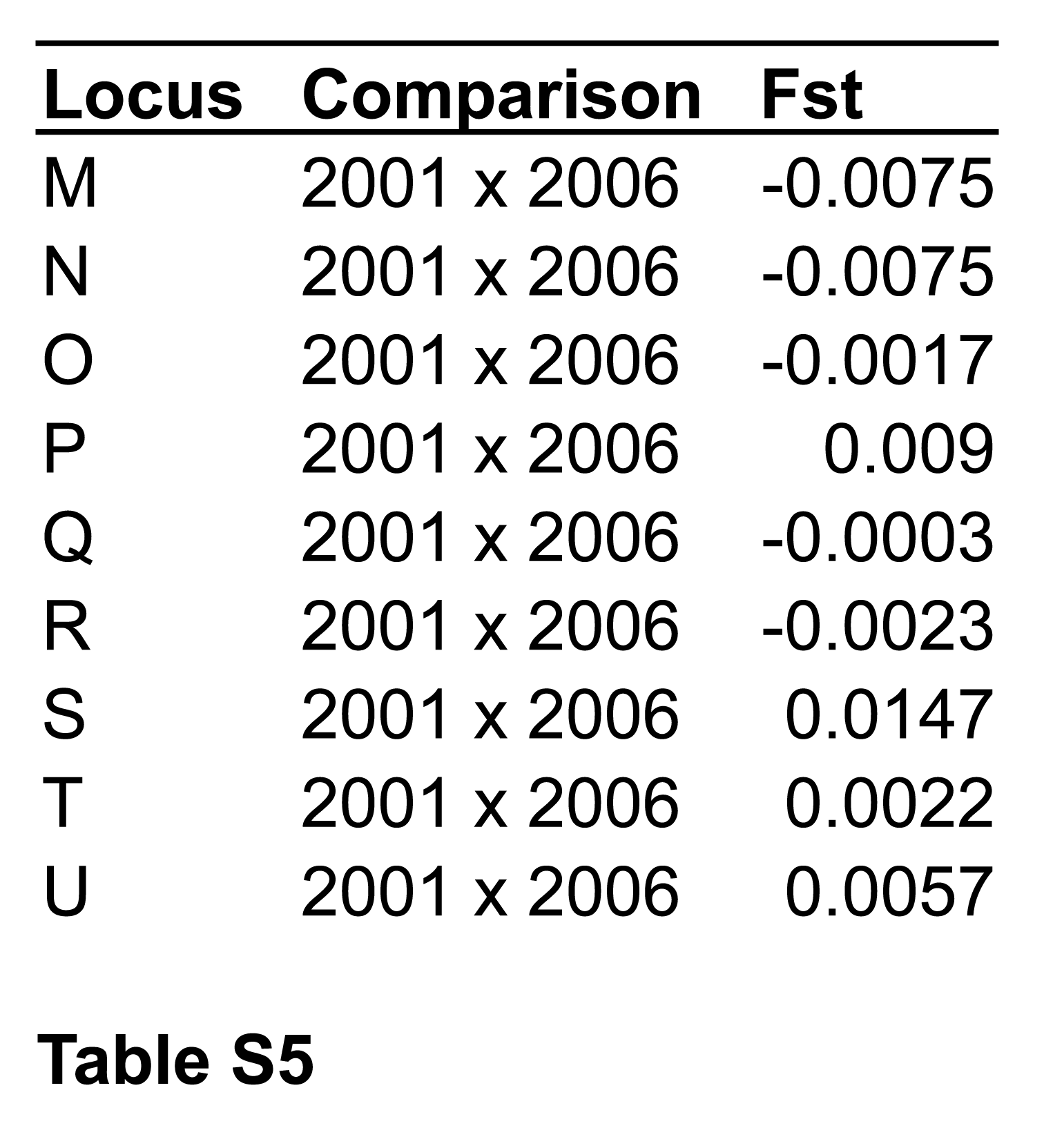

Supplement: S5 Table — FST analysis of differentiation between 2001 and 2006 Samoan population samples at the nine unlinked loci. (TIF) [file pgen.1004822.s008.tif]
