# Supplementary material for: The Evolution of Sex Ratio Distorter Suppression Affects a 25 cM Genomic Region in the Butterfly Hypolimnas bolina
Source: PLoS Genet. 2014 Dec 4;10(12):e1004822. doi: 10.1371/journal.pgen.1004822 (PMC4256269; doi:10.1371/journal.pgen.1004822)
Supplement: S1 Text — Modelling the spread of the suppressor. (DOCX) [file pgen.1004822.s011.docx]

**Text S1: Modelling the spread of the suppressor**

**The model**

We coded the model in Mathematica.

We model two linked loci, each with two alleles. The first locus can have the wildtype allele *s*, or the male-killing suppression gene *S*. The second locus is linked to the first, and has two selectively neutral alternative alleles denoted *A* and *a*. The model tracks the change in gametic frequencies from one generation to the next. There are four different basic gamete types: *AS*, *As*, *aS*, and *as*. Our individuals are diploid, so these four basic gamete types give nine possible basic individual genotypes: *AASS*, *AaSS*, *aaSS*, *AASs*, *AaSs*, *aaSs*, *AAss*, *Aass*, and *aass*.

The population is infected with *Wolbachia* at a frequency *J*. The infection is vertically transferred from mothers to offspring with 100% efficiency. Thus all of the offspring of an infected mother are infected, and none of the offspring of an uninfected mother are infected. Since there is this difference between males and females, we have to distinguish between male and female gametes. We also have to add infection status to the gametic genotypes to give a total of sixteen gamete types: four basic types, each from a male or female, and either infected or uninfected. Infection status and sex are also added to the individual genotypes to give thirty-six possible genotypes (the nine genotypes from above can be either infected or uninfected, and male or female). Note that for notational ease we still refer to these as “genotypes” although infection status is not a property of the individual’s genome.

The life cycle consists of mating, then selection. The gametic frequencies we begin with give rise to offspring genotypic frequencies. These offspring frequencies undergo selection, and thus result in adult genotypic frequencies. The adults produce gametes, which give us the gametic frequencies for the next generation. Along the way we can record the adult genotypic frequencies and consequently make predictions about the allelic frequencies observed in the real world.

In our model it is not generally the case that there are equal numbers of males and females, because *Wolbachia* affects the two differently. However, there are always equal numbers of male and female gametes (because each mating involves exactly one male and one female). Therefore the female gamete frequencies sum to one, and so do the male gamete frequencies.

Mating is assumed to be at random, and the mating step in our model consists of the transformation of the gametic frequencies into offspring genotypic frequencies. Given a female gamete at frequency *f*, and a male gamete at frequency *m*, the frequency of offspring resulting from combining these two gamete types will be the product of their frequencies *fm*. An added complication arises due to cytoplasmic incompatibility (CI). This is when mating between infected males and uninfected females result in fewer offspring than other matings. For simplicity, we assume that CI is total, so that fusions between infected male gametes and uninfected female gametes result in no offspring. We then renormalise the offspring frequencies appropriately so that they still sum to one for both males and females.

Once we have the offspring genotypes, selection occurs, transforming the offspring genotypic frequencies into adult genotypic frequencies. In our model, the only element of selection is male-killing: any infected males lacking the *S* suppressor gene (i.e. those with genotype *ss*) are killed by the *Wolbachia*. Males heterozygous at the suppressor locus (i.e. with the genotype *Ss*) are killed by their infection 50% of the time. Males homozygous for the suppressor (i.e. those with genotype *SS*) are not killed. For simplicity we assume no other selective effects. Notably this means that we are modelling the situation in which neither the suppressor nor the linked allele impose costs on their bearers.

Because females are unaffected by *Wolbachia*, there are now more females than males in our population. This will affect the observed allelic frequencies, since the allelic frequencies in males and females will differ (*s* genes, for example, will be more common in females because they are selectively neutral to a female, while males bearing *s* genes are more likely to die through male-killing). To account for this fact we renormalise the post-selection genotypic frequencies so that the sum of both male and female genotypic frequencies is one. This gives us the adult genotypic frequencies. From this data we get the model’s predictions for observed frequency of *A* and *S* alleles.

To complete the generation, we finally transform the adult genotypic frequencies into gametic frequencies. This is trivial in the case of most of the genotypes. However, for genotypes *AaSs*, things are more complicated. This is because *AaSs* individuals could have been formed by *AS* x *as* crosses, or by *As* x *aS* crosses. Denote the probability that a randomly-chosen *AaSs* individual was formed by an *AS* x *as* cross by *μ*, and the probability of recombination between the two loci of interest by *r*. Then the frequency of gamete types from infected *AaSs* individuals is

*AS*: ½(*μ*(1-*r*)+(1-*μ*)*r*)

*aS*: ½(1-*μ*(1-*r*)-(1-*μ*)*r*)

*As*: ½(1-*μ*(1-*r*)-(1-*μ*)*r)*

*as*: ½(*μ*(1-*r*)+(1-*μ*)*r*).

If we denote the probability that a randomly-chosen uninfected *AaSs* individual was formed by an *AS* x *as* cross by *ν* we can produce similar frequencies. It remains only to find *μ* and *ν*. But since we know the previous generation’s gamete frequencies this is trivial.

In the initial population of size *N* we suppose that the *A* and *S* alleles are absent. With a proportion *J* of the population infected with *Wolbachia*, the gamete types and frequencies in the population are therefore infected female *as* (frequency *J*), uninfected female *as* (frequency 1 – *J*), and uninfected male *as* (frequency 1). At this point the sex ratio is (1 - *J*)/(2 - *J*). We then introduce an immigrant of known sex and genotype into the population. To do this, we multiply the male gamete frequencies by *N*(1 - *J*)/(2 - *J*), and the female gamete frequencies by *N*/(2 - *J*) to give a “gamete mass” measurement. Then, given the immigrant’s genotype we get the probability that it produces a gamete of each type (we assume for simplicity that *μ* = *ν* = ¼). We then add the immigrant gamete probabilities to the gamete mass measurement, and renormalise so that female gamete frequencies sum to one, and so do male gamete frequencies. This gives us the initial gamete frequencies. As an example, in the case of a single infected male immigrant of genotype *AASS*, the initial gametic frequencies are:

Infected female *AS*: 0

Infected female *aS*: 0

Infected female *As*: 0

Infected female *as*: *J*

Uninfected female *AS*: 0

Uninfected female *aS*: 0

Uninfected female *As*: 0

Uninfected female *as*: 1 – *J*

Infected male *AS*: (2 - *J*)/(2 – *J* + (1 - *J*)*N*)

Infected male *aS*: 0

Infected male *As*: 0

Infected male *as*: 0

Uninfected male *AS*: 0

Uninfected male *aS*: 0

Uninfected male *As*: 0

Uninfected male *as*: (1 - *J*)*N*/(2 – *J* + (1 - *J*)*N*)

**Local LD**

Modelling local linkage disequilibrium is similar to above, with the added complexity that there are now two neutral loci to keep track of rather than just one. We label the alleles loci *A*/*a*, *B*/*b*, and *S*/*s* (the suppressor). Thus there are now 27 basic genotypes (3^3, since there are 3 loci, and three different possible genotypes at each one).

We now have to separately consider recombination between the suppressor and the two neutral loci (occurs at a rate *r*_1_), and recombination the two neutral loci (occurs at a rate *r*_2_). We make the simplifying assumption that these recombination events occur independently of one another, which is probably a reasonable assumption as long as the two rates aren’t too similar in size.

The final complication is that with so many more genotypes to keep track of, there are many more types of heterozygotes. In line with our calculation of *μ* and *ν* above, we have to calculate the frequency of each type of gamete being passed on by a given heterozygote.
